# Books Review

**Published:** 1871-04

**Authors:** 


### Books Review.

*On Diseases of the Spine and Nerves.* By Charles Bland Radcliffe, M. D., F. R. C. P. Lond., Physician to the Westminster Hospital, and to the National Hospital for the Paralyzed and Epileptic.—John Netten Radcliffe, Medical Superintendent of the National Hospital for the Paralyzed and Epileptic.—J. Warburton Begbie, M. D., F. R. C. P. Edin., Physician to the Royal Infirmary of Edinburgh.—Francis Edmund Ainstie, M. D., F. R. C. P., Senior Assistant Physician to Westminster Hospital; Lecturer on Materia Medica in Westminster Hospital School.—And John Russell Reynolds, M. D., F. R. S., F. R. C. P., Lond., Professor of the Principles and Practice of Medicine in University College; Physician to University College Hospital, and to the National Hospital for the Paralyzed and Epileptic. Philadelphia: Henry C. Lea. 1871.

We most heartily endorse the following publisher's notice: " This volume comprises a series of essays, extracted from the "System of Medicine," edited by J. Russell Reynolds, M. D., on a group of diseases of great interest, and many of them of frequent occurrence. These essays are from the pens of gentlemen of acknowledged ability and experience, who have paid particular attention to the several diseases on which they have written. The volume will be found to present the latest advances in our knowledge of the following subjects":

Diseases of the Spinal Cord; Meningitis; Myelitis; Spinal Congestion; Tetanus; Spinal Irritation; General Spinal Paralysis; Hysterical Paraplegia; Reflex Paraplegia; Infantile Paralysis; Spinal Hemorrhage; Non-inflammatory Spinal Softening; Induration of the Spinal Cord; Atrophy and Hypertrophy of the Spinal Cord; Tumor of the Spinal Cord; Concussion of the Spine; Compression of the Spinal Cord; Caries of the Vertebral Column; Spina Bifida; Epidemic Cerebro-spinal Meningitis; Neuritis and Neuroma; Local Paralysis from Nerve Disease; Local Sp sm; Torticollis; Local Anaesthesia."

---

*A Treatise on Physiology and Hygiene for Educational Institutions and general Readers.* Fully Illustrated. By JOS. C. HUTCHINSON. New York: CLARK & MAYNARD. 1870.

This work has remained on our table for several months, and has not until now received the attention it richly deserves. We have from time to time examined its contents and manner of arrangement, and have always been more and more strongly prejudiced in its favor.

The plain methods adopted for communicating knowledge to the young, and of attracting and interesting as well as instructing the student, has increased in special manner our admiration for the work as one eminently adapted to the wants of schools, academies and colleges. The text, while adapted to the popular wants, is at the same time full and complete, making it a book which maybe studied with profit by medical students, and those in quest of quite full knowledge of the subjects presented. It is very beautifully and fully illustrated, and cannot fail to commend itself in an eminent degree, to students and teachers of physiology.

---

*Health Officer's Annual Report of the City of Rochester.* By B. L. Hovey, M. D.

RELAPSING FEVER.

In this most excellent Report, we find the following upon Relapsing Fever and Disease of the Heart:—"Early after this disease appeared in New York, and published accounts of its prevalence were circulated, His Honor, the Mayor, called a special meeting of the Board of Health, and a committee was appointed to investigate its causes and to report to the board what was required to prevent the appearance in this city. At the request of the committee I corresponded with Dr. Harris, the corresponding secretary of the Metropolitan Board of Health, of New York City, upon the subject, and through his courtesy and gentlemanly kindness, I obtained from him the following, in answer to questions which I submitted, which may be inferred from the answers here given:

1. Relapsing Fever was not largely prevalent in the city of New York, for there were less than 500 cases in all since its outbreak, in October last.

2. That it is a malady of the poorest and most ill fed classes.

3. The exciting cause is dependent upon extreme poverty and the crowding together in ill ventilated apartments.

4. Relapsing fever is in a very high degree, communicable from sick to healthy, and the more confined the atmosphere in which sick and healthy are together the more certain is the disease communicated, and specially to those who have had poor or bad diet, or are intemperate.

5. Only two per centum die from this disease who are treated in the hospital and five per centum of those who are left at their lodgings.

6. It is difficult to diagnosticate relapsing fever from simple continued or Typhoid Fever in a single case, but easy after seeing a few cases or studying a single case for fourteen days.

7. The treatment is expectant and only varied from day to day, to meet symptoms as they appear.

8. The sanitary rules and regulations of the Metropolitan Board of Health are as positive and commanding as they are in Typhus Fever or Small Pox. Persons found sick with this disease are not allowed to remain in the apartments for a single day, and the premises, beds, clothing, &c., are not allowed to remain unpurified for even half a day.

The above is a brief of the answers given, and they are fully corroborated by published statements in medical journals by several of the leading physicians of New York, as well as by the best medical authority.

### DISEASE OF THE HEART.

This is reported to be a very common disease and is made the "cover of the causes" for many sudden deaths. It is not uncommon for persons to state that they have a "heart disease," and this supposition is confirmed by some pretender to *Medical Science*, with the consoling information that the sufferer is liable to die in a sudden and unexpected moment.

From such statements and teachings communities expect death in this sudden manner and attribute its cause to some organic disease of the heart. These same pretenders of medical science will look upon the dead body and with all the gravity of "*dignitaries*," say to friends and coroners, that "disease of the heart" was the cause of death. Such sayings are absurd and have a very injurious effect upon the living. It is much more honorable to say, we cannot tell the cause of death than to *guess at it*, for by doing that we affect whole families and make them distrustful of their own life and render them objects of fear and sadness through their whole existence.

Recent investigations of chronic disease of the heart. or lesions of that organ, fully show that sudden death is a very uncommon occurrence from that cause.

The present means of diagnosis of lesions of the heart are so perfect and can be discriminated with such accuracy, that there need not be any difficulty in diagnosing ordinary cases in life, and no physician is justified in guessing at it after death.

We may then positively assert that but few persons die suddenly of chronic disease of the heart, and the so often reported deaths from heart disease is no doubt made by persons or physicians who have not diagnosticated the case with any degree of accuracy.

---

*Comments of the Medical Press on the alleged Mal-practice Suit of Walsh vs. Sayre.*

We copy the following as showing the case very freely; and as it comes from England cannot be supposed to be influenced by considerations of favor:

"ALLEGED MAL-PRACTICE.—A gentleman well known by reputation in this country as an able surgeon, Dr. Sayre, of New York, has lately been subject to the annoyance of an unfounded action for malpractice, which was, we regret to say, supported by two of his medical brethren.

A child named Walsh, aged six years, was suffering from an abscess near the left hip. Dr. Sayre was called in, and in the presence of Dr. Gross and two other surgeons, punctured it, giving exit to a large quantity of pus.

The child's father, it is said, conferred with his "family physician," a person called Vaughan, who held a consultation with Dr. J. M. Carnochan and Dr. Willard Parker. They asserted that Dr. Sayre had punctured the joint, and allowed the synovial fluid to escape. An action to recover $20,000 was brought by the father. The case was, however, referred by the Supreme Court to three Referees—one, at least, of whom was a medical man. They found Vaughan was not a graduated physician, but had merely been employed in drug stores; that Drs. Carnochan and Parker had not made an examination of the alleged synovial fluid; that the patient had been treated with all proper skill and care; and that she had derived benefit from the operation. The Court confirmed the report, allowing costs to Dr. Sayre. The plaintiff again brought the case before the Supreme Court, by moving that the defendant should show cause why one of the Referees—Dr. Swinburne—should not be set aside on the ground of incompetence. This was refused with costs.

Assuming the correctness of the narrative from which the preceding abstract has been taken, we must say that the action appears to have been a most disgraceful one; and that the conduct of Drs. Carnochan and Parker—who, we believe, are men of some standing in their profession in America—was, to say the least, very reprehensible. The New York *Medical Journal*, in noticing the case, makes a remark with which we heartily agree:

" We have but a single regret to offer in view of the happy termination of " the suit, and that is, that those who instigate such proceedings could not be " made to suffer an equally severe penalty with that which they would extort " from their designed victims."—*British Medical Journal.*

---

*Medical and Surgical Memoirs.* By JOSEPH JONES, M. D., Professor of Chemistry, Medical Department, University of Louisiana.

This work will embrace the investigations of Fifteen Years into the Causes, Geographical Distribution, Natural History and Treatment of Intermittent, Remittent and Congestive Malarial Fevers, Yellow Fever, Typhoid and Typhus Fevers, Small Pox, Spurious Vaccinnation, Measles, Pneumonia, Diarrhœa, Dysentery, Scurvy, Tetanus, Cerebro-Spinal-Meningitis, Diseases supervening upon Gun-Shot Wounds, Pyœmia, Hospital Gangrene, Erysipelas, etc.

The result of the investigation of the Diseases of the Confederate Army during the American Civil War, 1861-1865, will occupy a prominent portion of the work.

These investigations have been prosecuted unremittingly during the past 15 years; and the author proposes to lay the results before the Medical profession, as soon as a sufficient number of subscribers have been obtained.

Physicians and others desiring to become subscribers, will please forward their names; and those receiving this Circular are respectfully requested to call the attention of their friends, and also of the County and State Medical Societies to the proposed work.

There will be two volumes of 1000 pages each, furnished to subscribers at actual cost.

Address, Joseph Jones, M. D., Glass Box 1542, New Orleans, La.

---

*Treatment of Croup.* By FORDYCE BARKER, M. D., with Remarks by A. JACOBI, M. D.

The careful reader of this very instructing pamphlet, is liable to conclude that as yet we *know* nothing of any great value, as to the nature, defferential diagnosis or treatment of this most common and formidable disease. There is yet no harmony of opinion as to the nature and varieties of the affection, and no uniformity in treatment, the whole being based upon some supposed indication, and for the most part treated with medicines of questionable value. When authors announce uniformly favorable results in their treatment of croup, during a period of twenty years, physicians who have seen much of the disease, and observed its fatal effects, naturally conclude that they exclude from their nomenclature all cases which prove fatal, thus obtaining a great success in disease regarded by others as generally fatal.

This pamphlet is worthy of careful perusal: is a reprint from the American Journal of Obstetrics, published by W. A. Townsend & Adams, New York.

---

*Guide to the Examination of the Urine.* By J. WICKHAM LEGG, M. D. Second Edition. Philadelphia: LINDSAY & BLACKINSTON. 1870.

This little work, we believe, is well adapted to supply the student of medicine a concise guide in examinations of the urine. A plan is given, step by step, by which to determine the nature of the alterations which most frequently occur in disease. Its condensed form permits examination by the most busy practitioner, and we believe it will prove a valuable guide in the examinations which are so frequently required.

---

*Circular Nos. 3 and 4.* War Department, Surgeon General's Office—Report on Barracks and Hospitals, with descriptions of Military Posts, and approved Plans and Specifications for Post Hospitals.

These reports are very instructive and valuable, as indeed are all the publications from the Surgeon General's Office. The reports and plans constitute a standard work upon the subject which will be appreciated by those who direct the construction of Military Forts, Hospitals, &c.

The energy and ability with which the Surgeon General's Office furnishes valuable medical, surgical and sanitary reports, is creditable alike to the office and country, to say nothing of the individual merit which is every where apparent. It is the more noticeable since it makes such striking contrast with the same office before the war. Truly war is not an unmixed evil—out of it may spring some valuable knowledge.

*Report of the Board of Health of the city of Chicago for* 1867, 1868 *and* 1869; *and a Sanitary History of Chicago from* 1833 *to* 1870. Lakeside Publishing and Printing Co., Chicago.

This volume contains a vast amount of Sanitary and Hygienic science and literature, together with valuable historic record of the most rapidly grown, great city of the world. It has charts and maps showing the points of greatest prevalence of cholera and other epidemic disease, the text pointing out the possible causes of such prevalence.

The report is very creditable to the Board of Health from which it originates, and will prove interesting and instructive for future reference.

---

*Retention of Urine dependant on Stricture of the Urethra.* By ALEXANDER STEIN, M. D.

This paper was first read before the New York Journal Association. It is now published in pamphlet form, and is an excellent lecture on Stricture.

---

*Photographic Review of Medicine and Surgery.* Edited by F. F. MAURY, M.D. and L. A. DUHRING, M. D. Published by LIPPINCOTT & Co., Philadelphia.

This is an attractive Journal of Medicine and Surgery, teaching and illustrating cases in the most natural and accurate mode, "What we see we know." It is published bi-monthly at $6.00 in advance. It is worthy of support by the profession. The plates are large and life-like—true as nature itself.

